# PedaleoVR: Usability study of a virtual reality application for cycling exercise in patients with lower limb disorders and elderly people

**DOI:** 10.1371/journal.pone.0280743

**Published:** 2023-02-22

**Authors:** Ana Rojo, Arantxa Castrillo, Cristina López, Luis Perea, Fady Alnajjar, Juan C. Moreno, Rafael Raya

**Affiliations:** 1 Departamento de Tecnologías de la Información, Escuela Politécnica Superior, Universidad San Pablo-CEU, CEU Universities, Madrid, Spain; 2 NeuroRehabilitation Group, Cajal Institute, Spanish National Research Council, Madrid, Spain; 3 Werium Assistive Solutions, Madrid, Spain; 4 Centro Lescer, Madrid, Spain; 5 Albertia Servicios Sociosanitarios, Madrid, Spain; 6 Department of Computer Science and Software Engineering, College of IT, United Arab Emirates University, Al Ain, UAE; Prince Sattam Bin Abdulaziz University, College of Applied Medical Sciences, SAUDI ARABIA

## Abstract

Achieving adherence to physical exercise training is essential in elders and adults with neurological disorders. Immersive technologies are seeing wide adoption among new neurorehabilitation therapies, as they provide a highly effective motivational and stimulating component. The aim of this study is to verify whether the developed virtual reality system for pedaling exercise is accepted and could be safety, useful and motivating for these populations. A feasibility study was conducted with patients with neuromotor disorders and elderly people from Lescer Clinic and the residential group Albertia, respectively. All the participants performed a pedaling exercise session with virtual reality platform. Then, the Intrinsic Motivation Inventory, the System Usability Scale (SUS), Credibility and Expectancy Questionnaire, were assessed in the group of 20 adults (mean age = 61.1; standard deviation = 12.617, 15 males and 5 females) with lower limb disorders. While the Simulator Sickness Questionnaire, Presence Questionnaire, Game user Experience Satisfaction Scale and SUS were assessed in the group of 18 elders (mean age = 85.16; standard deviation = 5.93, 5 males and 13 females). In light of the outcomes, PedaleoVR is considered to be a credible, usable and motivational tool towards adults with neuromotor disorders to perform cycling exercise, and therefore its usage could contribute to adherence to lower limb training activities. Moreover, PedaleoVR does not generate negative effects related to cybersickness while the sensation of presence and the degree of satisfaction generated have been positively evaluated by the geriatric population. This trial has been registered at ClinicalTrials.gov under the identifier: NCT05162040, Dec 2021

## Introduction

The most frequent causes of sudden neurological injuries and lower limb disorders (LLD) are trauma and stroke [1]. Regarding the rehabilitation of locomotion after spinal cord injury or stroke, there has been considerable controversy and debate about the efficacy of the different approaches used [2]. New approaches propose an adaptation of therapy to the patients’ motor learning process [3–5]. Although the referenced literature focuses on the rehabilitation of cerebrovascular patients, due to the extension of this field, the idea of adapting therapy to the motor learning process can be equally applied to patients with other neurological lesions in a way that therapeutic exercises are combined with stimulating environments.

According to the statistics of the Eurostat, “in 2019, more than one fifth (20.3%) of the European Union (EU) population was aged 65 and over” [6]. Moreover, the growing pace of elderly segment population is concerning, since the EU population over 80 years is projected to increase from 5.8% to 14.6% to 2100 [6]. As it is well-known, population undergoing an aging process eventually suffers a series of neurophysiological events that affect the loss of muscle mass, strength and balance control, causing falls in the elderly [7]. Often, those who suffer a fall must undergo long periods of rehabilitation to get full recovery, affecting their dependence in their daily lives. Indeed their functional situation has an impact on the quality of life of these patients [8]. With the determination to reduce the rate of falls, several scientific studies agree that physical exercise can help to attenuate the incidence of the so-called age-related conditions [9]. More effective interventions based-on personalized exercises for the patient and designing physical training programs can improve the muscle strength and balance, alleviating the decline in mobility in the elderly [10].

For both populations, patients with LLD and elderly people, a common interest is identified: the need for training tools to encourage physical activity to improve motor control, stability in gait function and lower limb strengthening. Regarding regular physical activity (PA) therapies for these populations, there are more and more PA interventions that propose pedaling activities, since the use of exercise bikes presents an affordable cost for patients and they are simple to use [11]. However, most of the programs that promote pedaling as a practice for physical strengthening do not offer real-time progress information to clinicians due to the lack of standard definition, follow-up protocols and quantifiable indices of functional improvement [12, 13]. On the other hand, in terms of emerging technologies applied to this area, immersive technologies stand out. Their potential lies in the ability to generate controlled and personalized immersive environments where the movements made by the patient can be captured and objectively quantified. Through immersive environments to the therapy makes potential motor learning a transparent process for the user. Moreover, modifying different sensory aspects of the learning environment can influence motor behavior [14]. In the course of therapeutic programs, adding simple sensory stimulation could improve sensory and motor function in neurological patients [15]. Latest studies have focused on demonstrating that stimuli environments-based physical training facilitates the recovery of motor function in neurological patients [16, 17]. Exergames technologies, such as Nintendo Wii™or Kinect™, are widely used to stimulate older adults in initiating or maintaining physical activity [18]. These technologies have had a rapid adoption in this field due to its low-cost, but also they are relatively simple to install and use [19, 20]. In addition, exergames with computers as well as virtual reality clearly provides a positive motivational aspect for physical activity [18, 20]. These rehabilitations based on immersive technologies increase patient motivation by allowing to perform physical activities in virtual environments (VEs), providing the patient feedback on the goals achieved. All these strategies are based on task repetition, which increases intensity and tension during exercise and facilitates motor learning and neuroplasticity [21].

To the authors’ knowledge, even the use of these interactive technologies does not always guarantee better outcomes in PA by themselves, it can be assumed that they enhance adherence to training programs and physical activity, which has a positive effect on motor functioning on the older adults and patients with LLD. The importance of motivation itself in every physical field is undeniable, but in neurorehabilitation, the use of interactive technology and exergames-based system have proven to be effective to motivate persons with disabilities to perform exercise [22]. But whether the use of these technologies is fully accepted by this population more than other tools to perform physical exercise and how motivating are they perceived is being explored recently by some authors [22–24]. And their conclusions call for more evidence to support their tentative conclusions. The researchers of this study developed a novel virtual reality platform designed to achieve greater adherence to cycling exercise through the use of gamification strategies and user motivation [25] for adults with LLD and older adults. Due to these good results in the technical validations, the researchers understand that the developed tool will be used by the population of people with motor disorders if they find it motivating and useful for their rehabilitation process, while it will be used by the adult population if they positively tolerate this technology and are satisfied with its use. Thus, the researchers wish to validate the following hypotheses:

- Do patients with LLD see this virtual reality platform for pedaling as a positive value for their rehabilitation? Does the use of this virtual platform provide them with motivation for physical exercise pedaling?
  To address these questions we hope to gain information on credibility and intrinsic motivation ratings.
- Do adults accept virtual reality technology as a tool for pedaling physical activity? Are they satisfied with using this platform?
  To address these questions we expect to gain information on satisfaction, sense of presence, and user experience ratings.
- Do both populations find the platform design easy to use? To address this question we hope to validate the tool from a usability point of view.

In general, it is expected to validate the characteristics of virtual reality in this platform for the promotion of the approximate pedaling activity from two different populations that could find in its use a potential benefit. Then, in the present study, the differences between the two populations are known and respected by the authors. The intention is not to compare them but to evaluate how the same VR platform for the promotion of the pedalling activity can nurture relevant aspects of use for each population. Therefore, the data from each case are shown separately throughout the Results and Discussion sections.

## Materials and methods

### Participants

The participant screening protocol was based on the following inclusion and exclusion criteria applied by the physicians of the Lescer Clinic and Albertia. Inclusion criteria were: (1) individuals were eligible if they had been prescribed pedalling exercise as treatment for lower limb training or rehabilitation (2) They also had to be able to perform a pedalling session with virtual reality technology.

Exclusion criteria were: (1) an insufficient cognitive state, in particular, presence of dementia or mild cognitive impairment; (2) an unbound bone fracture; (3) severe disorders of vision and/or audition (inability to perceive visual and/or auditory information coming from virtual reality); (4) whose clinical record ruled out any incompatibility with the use of a virtual reality system.

**Fig 1:**
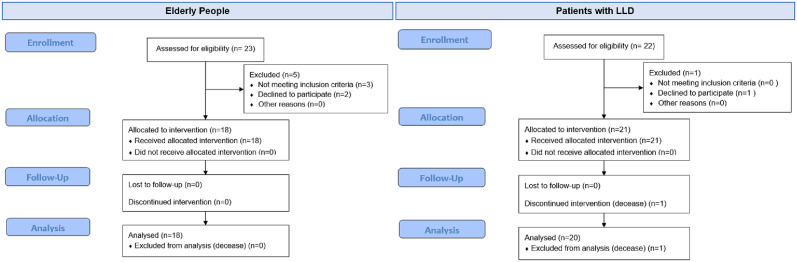
CONSORT flow diagrams of the elderly people from Albertia Servicios Sociosanitarios and patients with lower limb disorders from Lescer Clinic.

The CONSORT diagram (Fig 1) shows the participant flow through the study, including enrollment, experimental intervention and analysis. As it is depicted in Fig 1, finally eighteen elder participants from Albertia met these criteria (5 males and 13 females, mean aged = 85.16 (standard deviation = 5.93)) and provided written consent to be enrolled onto the study. Likewise, 21 participants from Lescer Clinic met these criteria and provided written informed consent to be enrolled onto the study, but only 20 participants completed the study (15 males and 5 females, mean aged = 61.10 (standard deviation = 12.62)). Participants with neurological pathologies were diagnosed with (6) ischemic strokes, (1) hemorrhagic stroke, (1) thalamic stroke, (1) internal capsule stroke (3) traumatic brain injury (TBI), (1) Parkinson syndrome, (1) mixed axonal neuropathy with sensory demyelination, (1) progressive multifocal leukoencephalopathy, (1) secondary obstructive hydrocephalus, (1) angioma avernosus hemorrhage, (1) hemiprotuberancial hemorrhage—cavernoma, (1) ataxia and (1) cerebral artery aneurysm. The clinical conditions, gender and age of the participants are shown in Table 1.

**Table 1:** Socio-demographic and clinical characteristics of patients from Lescer Clinic by gender, age and clinical condition.

Gender	Age	Clinical Condition
M	59	Hemorrhagic stroke
M	39	Thalamic stroke
M	75	Traumatic brain injury
M	45	Cerebral artery aneurysm
F	71	Ischemic stroke
M	57	Ischemic stroke
F	88	Ataxia
M	71	Internal capsule stroke
M	39	Severe traumatic brain injury
M	62	Ischemic internal carotid stroke
M	77	Axonal mixed neuropathy with sensory demyelination
F	64	Angioma avernosus hemorrhage
M	53	Ischemic stroke
M	53	Ischemic stroke
M	62	Ischemic stroke
M	58	Hemiprotuberancial hemorrhage—cavernoma
F	72	Traumatic brain injury
M	56	Progressive multifocal leukoencephalopathy
M	72	Parkinson syndrome
F	49	Ischemic stroke

### Procedure

All the participants gave written informed consent, in accordance with the Research Ethics Committee of Universidad CEU San Pablo (approval code: 550/21/51). Additionally, the protocol of the study was registered at Clinicaltrials.gov with reference: NCT05162040. The privacy rights of human participants were observed at all times.

Prior to starting the pilot and completing the questionnaires, written informed consent was obtained, and the participants read instructions of the questionnaires.

First, a practical explanation of familiarization with the instrumentation is carried out, aimed at acquiring basic skills in the use of the virtual reality environment synchronized with the pedaling task. The ‘Landscape Flight’ scenario was used for the familiarization trial since it is the most peaceful VE of all three and it has fewer distracting elements. Then, a pedaling exercise is performed on a static pedaling ergometer synchronizing the physical activity with the visual feedback of the virtual reality application. Two pedaling sets of 5 minutes each are performed, with 1 minute rest between sets. All the participants underwent both sets of pedalling with the ‘High Flight’ VE. This scenario infuses greater sensation of dynamism due to the displacement of the clouds, the speed of the plane’s movement and the speed of the propeller. Moreover, the appearance of animated elements such as birds, other planes and fog banks, along the route, are elements that can captivate the user’s attention, increasing their sense of presence. Once the exercise task was completed, several questionnaires were administered by a researcher to evaluate the experience of the patients with LLD and the elderly participants with PedaleoVR. In the case of the elderly, the cybersickness survey was taken before and after the use of PedaleoVR. All responses of all the questionnaires were subsequently digitized and the paper questionnaires filed.

### Virtual reality cycling platform

A lower extremity motor training platform has been developed for adult patients with impaired control due to neurological damage and older adults using an immersive virtual reality system that establishes a progressive and individualized training program based on the rehabilitation of gait function.

PedaleoVR implements extrinsic feedback strategies, gamification by levels, and personalising of the sessions with the aim of achieving greater adherence to the users’ pedaling exercise sessions. Its immersive nature means an increase in the feeling of “presence”, generating an impact on the subject’s involvement in achieving the training objectives.

#### Description of the VR platform: PedaleoVR

PedaleoVR consists of two parts: a sensing system which integrates micro-controller unit (MCU), inertial measurement unit (IMU) and a Bluetooth module, and a virtual rehabilitation training scene, as shown in Fig 3. This VR system is based on the communication of pedaling data and surrounding information to the control computer. The data transmission from the inertial sensors to the Oculus Quest 2 head-mounted display (HMD) is established via Bluetooth. The VR platform supports the data processing of pedaling cycles, speed and distance traveled of each user and the transmission of these values to the immersive scenarios.

The motion capture system for pedal kinematic analysis to be used is the ENLAZA^™^ sensor from Werium Assistive Solutions, due to the proven reliability of its ROM measurements at the cervical [26], wrist and elbow joints [27]. The ENLAZA^™^ sensor module contains an inertial measurement unit (IMU) with 9 degrees of freedom, which integrates a 3-axis accelerometer, a 3-axis gyroscope and a 3-axis magnetometer. The sensor also includes a Bluetooth module (2.4 GHz) through which the IMU data is sent to the virtual reality device.

#### Virtual scenarios

**Fig 2:**
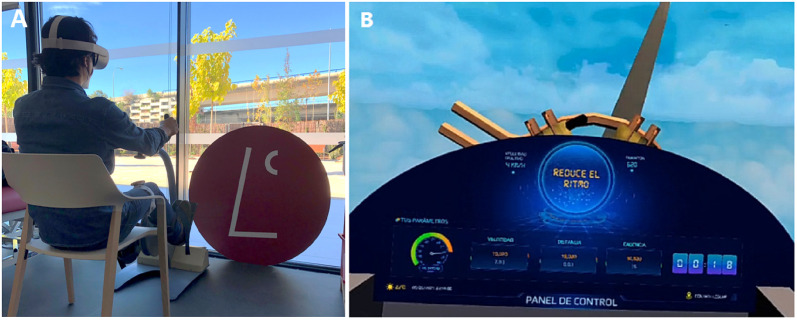
Pilot session with subjects with neuromotor disorders. (A) Participant using the virtual cycling platform. (B) Capture of the first-person view of the virtual scenario ‘High Flight’.

The PedaleoVR was developed using Unity3D Game Engine software. In total, 3 virtual games were developed. These VR scenarios generated for this therapy consists of controlling the forward movement of a vehicle by pedaling. Thus, the user is placed inside the vehicle’s cabin and visualizes the session data on the control panel (Fig 2).

**Fig 3:**
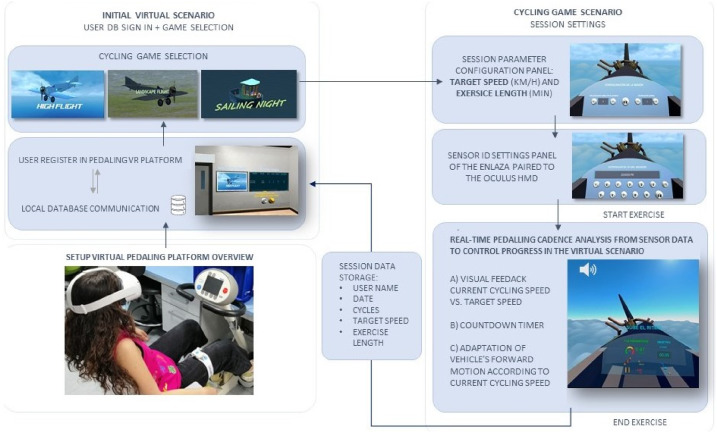
Workflow diagram of the Pedaling Virtual Platform user experience. MOTOmed scheme [28].

The whole virtual platform consists of two main spaces. Firstly, there is a standby area where the user logs in him/her-self in the system, his/her ranking records are displayed and he/she can select the game ambience where to perform the pedalling session. The next space of this experience consists of the three games with different ambience scenery (see Fig 3). (i) **Game High Flight**: the navigation vehicle is a light aircraft and the flight environment is the sky; (ii) **Game Landscape Flight**: the navigation vehicle is a light aircraft and the flight environment is a canyon valley; (iii) **Game Sailing Night**: the navigation vehicle is a fishing vessel and the sailing environment is the sea.

#### Visual biofeedback

The system implemented in the VR platform evaluates every second the average pedaling speed of the last 3 seconds with respect to the target speed. A threshold of acceptance of the instantaneous speed is set at ±15% of the target speed. Higher values are considered too fast and lower values too slow, so pop-up messages are generated to moderate or increase the pedaling cadence accordingly. Motivational messages are displayed when the user maintains an adequate pace.

### Measurements

All assessments were performed by the researchers of the study. The following 6 questionnaires were used for the corresponding assessments of each group of participants, neurological patients and elderly people. The datasets used and/or analysed during this study are available at the address specified in the Supporting Information Captions section.

#### Intrinsic motivation inventory

The IMI is considered a reliable assessment (intraclass correlation = 0.70) and was selected to evaluate the motivation to use the PedaleoVR. It assesses the participant’s subjective experience related to a target activity in laboratory experiments, in this case the PedaleoVR exercises. The instrument assesses participants interest/enjoyment, perceived competence, effort, value/usefulness, felt pressure and tension, and perceived choice while performing a given activity, thus yielding six subscale scores. The IMI items have often been modified slightly to fit specific activities. Nonetheless, shorter versions have been used and been found to be quite reliable. The present study used the IMI 25-item version which includes the three subscales of value/usefulness, interest/enjoyment, and perceived choice. A total IMI score is not recommended, therefore subscale scores, each with a recalculated maximum score of 7, are used in the analyses [29].

#### Credibility and expectancy questionnaire, CEQ

The CEQ was selected to evaluate the credibility and expectancy with regard to the PedaleoVR for improvement of PA and is also considered a reliable assessment (Cronbach’s alpha = 0.85). The Credibility/Expectancy Questionnaire is the most widely used measure of treatment credibility and expectancy in psychotherapy research. It contains 6 items rated on a 1–9 or a 0%-100% scale, depending upon the item. This revised scale, which was used in the present study, has been subjected to factor analysis, with results indicating that the items load onto two distinct factors of credibility and expectancy. The first three items of the scale load onto the credibility factor and the final three items load onto the expectancy factor. The maximum score on each subscale is 27. A score of 13.5 is considered neutral, everything above 13.5 is positive while everything under 13.5 is considered negative [30].

#### Simulator sickness questionnaire, SSQ

The SSQ is widely used in VR research to assess users’ level of sickness symptoms based on subjective severity ratings of 16 symptoms on a scale from 0 (no perception) to 3 (severe perception) after the exposure [31]. The ratings for individual symptoms are divided into three non-exclusive categories that represent symptoms of nausea (N), oculomotor disturbance (O), and disorientation (D). The formulas dictate that the sum of nausea, oculomotor disturbance and disorientation, are multiply by the scaling factors 9.54, 7.58 and 13.92, respectively [31]. While the total simulator sickness score (TS) is computed by multiplying the sum of each category by the scaling factor 3.74. Therefore, a SSQ total scores above 20 is considered “bad” [32]. Similar thresholds can be assumed for the sub-scales nausea, oculomotor disturbance, and disorientation as the scaling factors were chosen to produce scales with similar variations [31].

#### Presence questionnaire, PQ

VR studies commonly use the Witmer and Singer (1998) Presence Questionnaire (PQ) [33]. We used PQ Vs. 3.0, Nov. 1994, revised by UQO Cybersecurity Lab in 2004 which has been widely tested for reliability. PQ includes 24 questions that measure factors such as realism, control, quality of interface, possibility to examine, possibility to act, self-evaluation, sounds and haptic. Since in our study was not possible to manipulate objects with and did not include sounds, the optional sound and haptic questions were excluded, resulting in a 19-questions survey. Each question is evaluated on a 7-point Likert scale.

#### Game user experience satisfaction scale, GUESS

To assess the satisfaction of gamified virtual application, we used the 18-item short scale of the Game User Experience Satisfaction Scale (GUESS-18) [34]. This questionnaire is a brief, practical, and comprehensive measure of video game satisfaction for practitioners and researchers, which is recommended to use in iterative game design, testing, and research. The GUESS-18 scale consists of nine subscales: usability/playability, narratives, play engrossment, enjoyment, creative freedom, audio aesthetics, personal gratification, social connectivity, and visual aesthetics. The GUESS-18 items are rated with a 7-point Likert scale (1 = Strongly Disagree to 7 = Strongly Agree). Calculating the subscales scores of the GUESS-18 consists of averaging the items in that subscale and an overall score calculated by summing the subscale scores.

#### System usability scale, SUS

The SUS test has become an industry standard as it allows to evaluate a wide variety of products and services, including hardware, software, mobile devices, websites and applications. Whereby, the SUS was selected to evaluate the usability of PedaleoVR within adults and is also considered a reliable assessment (Cronbach’s alpha = 0.91). The item scores on the SUS range from 1 (totally disagree) to 5 (totally agree) and are converted into a score from 0 (negative) to 100 (positive). A score of 72.5 or higher is considered good and above 85.0 is excellent [35].

### Statistical analysis

To determine the sample size for these feasibility studies of PedaleoVR in different populations, we set the following hypothesis: We want to identify whether these issues (usability, credibility, intrinsic motivation, sense of presence, VR sickness and satisfaction) affect 10% or more of our participants with an 85% probability of detecting them in a feasibility test. With these requirements, we need to recruit at least 18 participants, which is estimated from the formula: log(1-.85) / log(1-.10) = 18.006. Therefore, the samples of 18 and 20 participants were presumed appropriate to capture heterogeneous data for analysis. In this paper, means and standard deviations were calculated for each of the metrics. These parameters allow us to statistically describe the aspects of credibility, expectation, intrinsic motivation and usability for the population of patients with LLD, and the aspects of satisfaction, sense of presence, generation of adverse aspects and usability for the population of older adults. Descriptive analysis of the questionnaire results and graphic plots were computed and generated with IBM SPSS Statistics (version 27.0).

## Results

**Fig 4:**
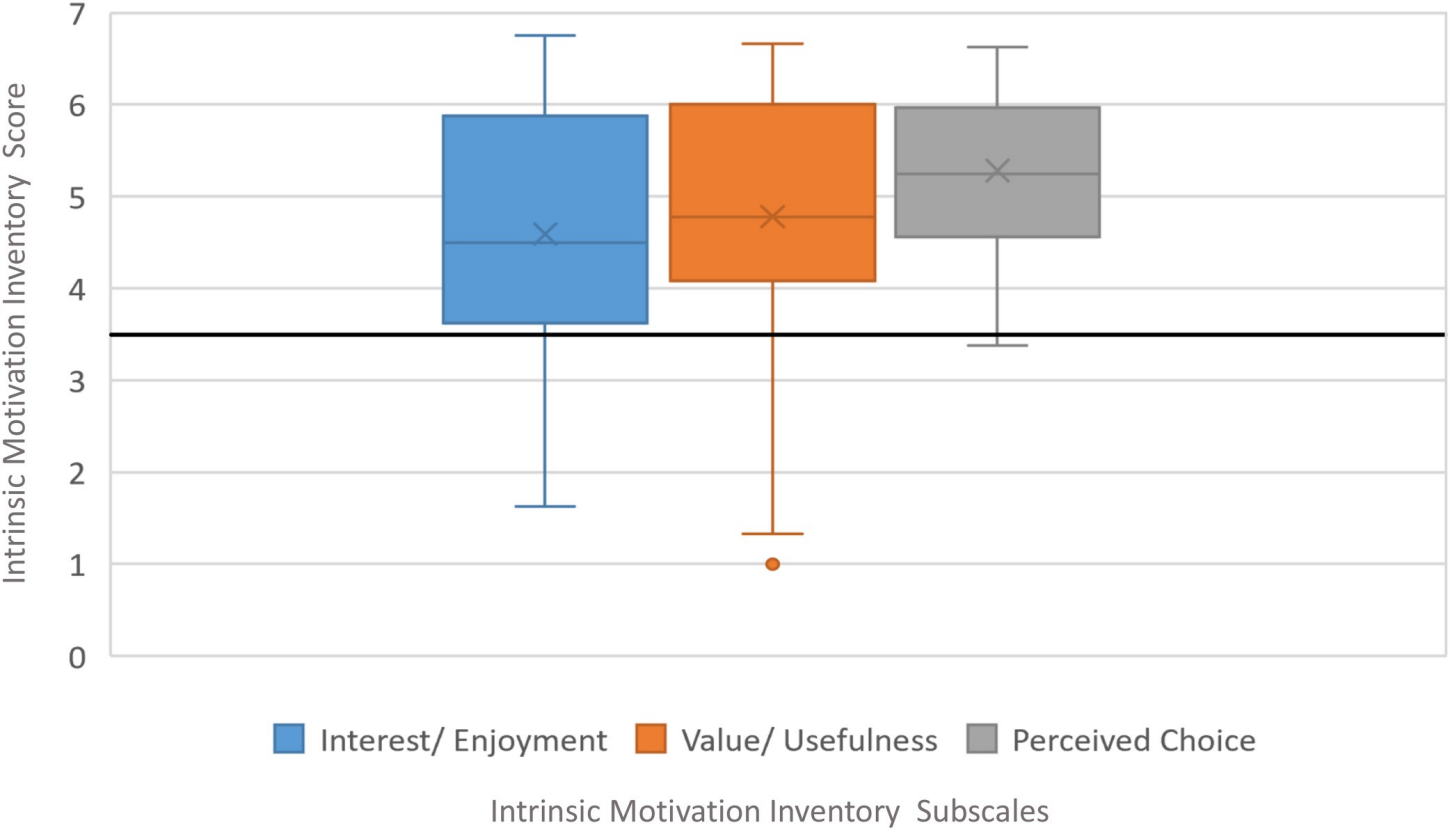
Boxplot distribution of IMI assessment: Interest/Enjoyment, Value/Usefulness, perceived choice.

**Fig 5:**
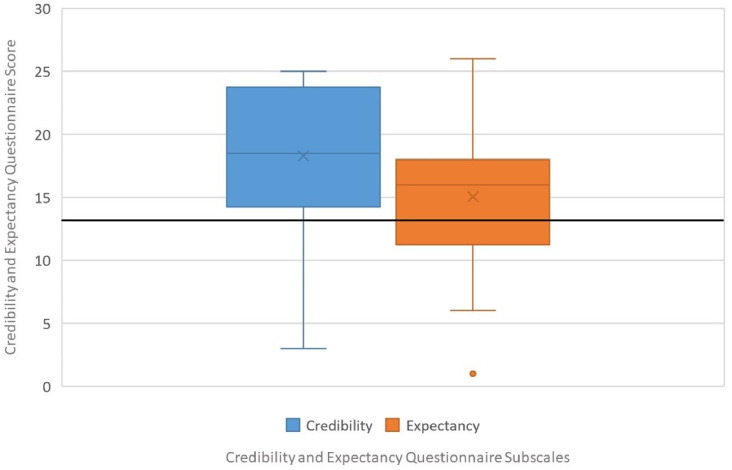
Boxplot distribution CEQ assessment: Credibility and expectancy.

**Fig 6:**
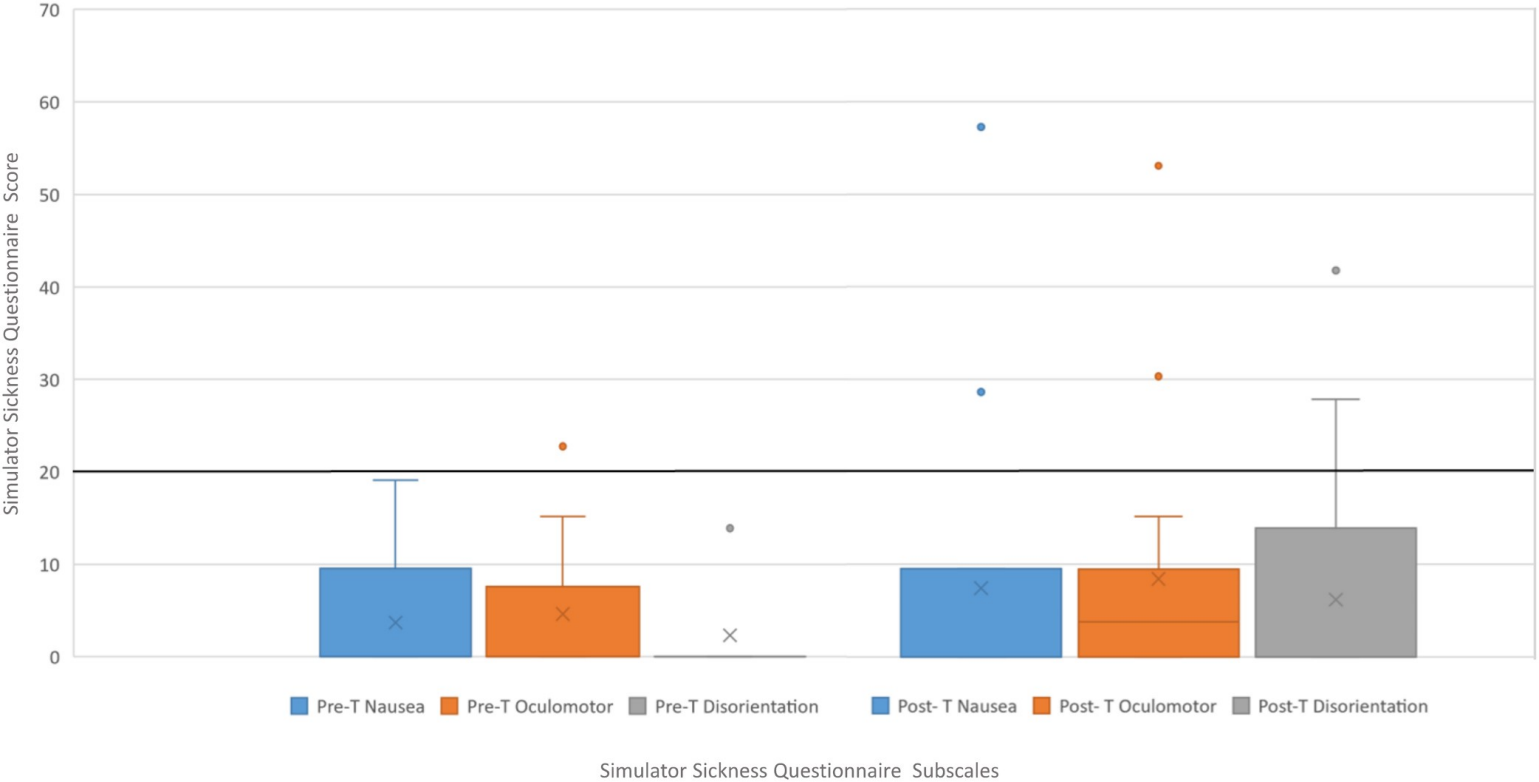
Boxplot distribution of Pre-Post SSQ assessment.

**Fig 7:**
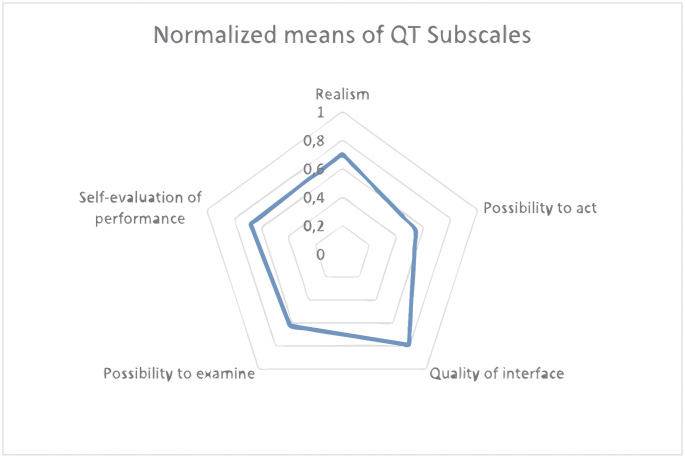
Radar plot of normalized means of PQ subscales assessment.

**Fig 8:**
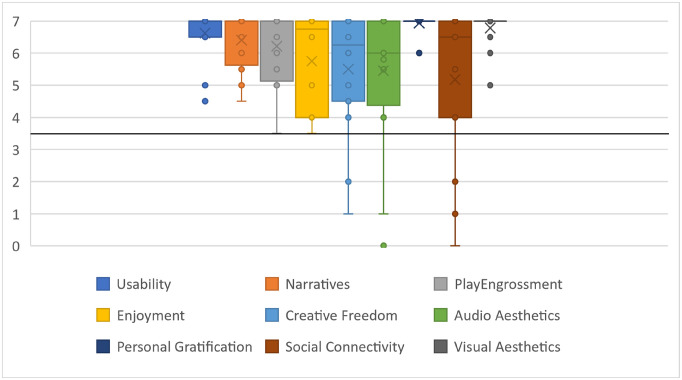
Boxplot distribution of GUESS-18 subscales.

**Fig 9:**
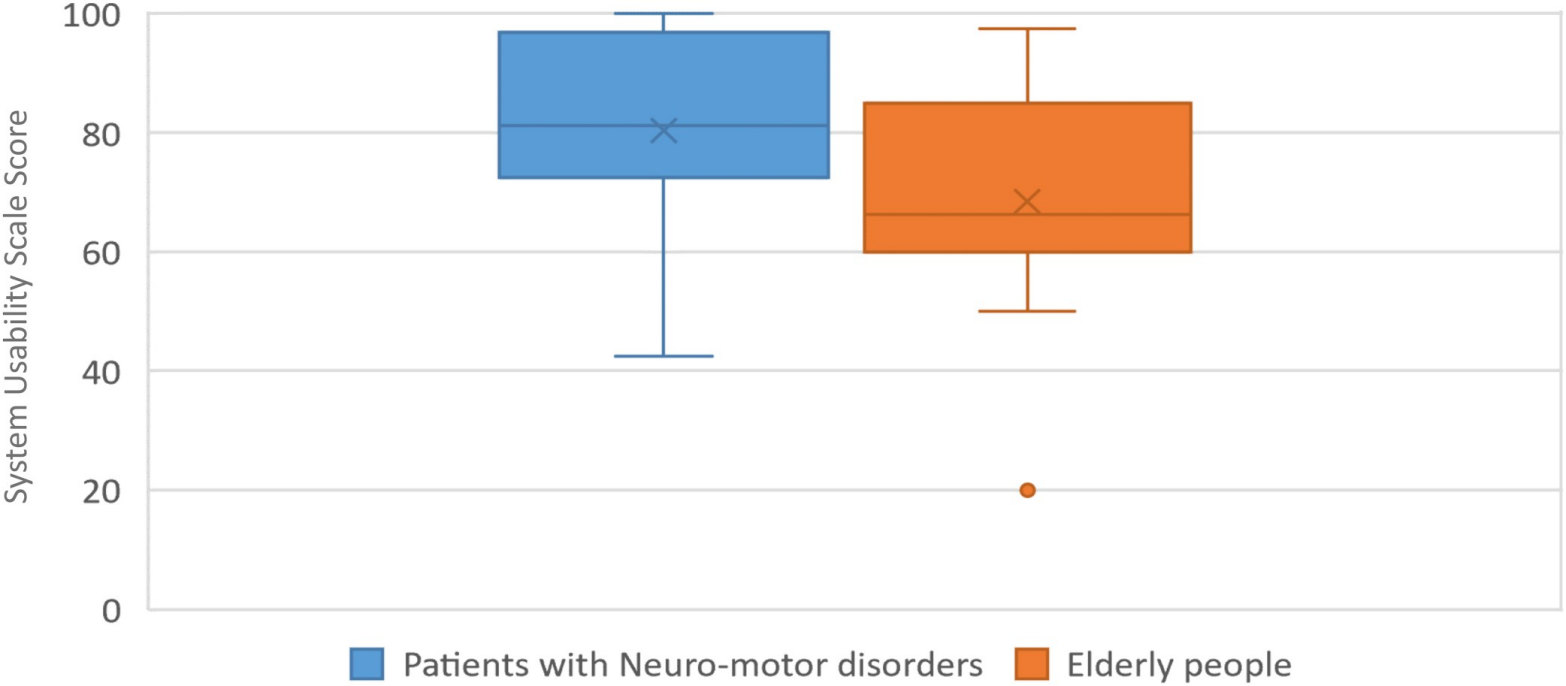
Boxplot distribution of SUS assessment of both groups.

Table 2 includes the descriptive analysis of the IMI, CEQ and SUS questionnaire responses of patients with LLD. IMI mean values of each subscale are shown in Fig 4, and CEQ mean values of each subscale are shown in Fig 5. The results of cybersickness questionnaire of older adults are included in Table 3 and the cybersickness ratings of the previous-exposure and post-exposure are shown in Fig 6. Table 4 includes the outcomes of presence, satisfaction and usability of older adults. Regarding the PQ outcomes, the mean values obtained in each subscale are shown normalized in Fig 7. GUESS-18 mean values of each subscale are shown in Fig 8. And the SUS outcomes for both groups are shown in Fig 9.

**Table 2:** Mean and standard deviation (SD) outcomes of Intrinsic Motivation Inventory (IMI), Credibility/Expectancy Questionnarie (CEQ) and System Usability Scale (SUS) of patients with neuromotor disorders. The interpretation of the mean scores for each subscale is provided in the form: U = unacceptable outcome; A = acceptable outcome; HD = highly desirable outcome.

Assessment	Mean (SD) (n = 20)	Score interpretation
**IMI**		
Interest/Enjoyment	4.593 (1.363)	U = 0–3; A = 3–5; HD = 5–7
Value/Usefulness	4.783 (1.555)	U = 0–3; A = 3–5; HD = 5–7
Perceived Choice	5.281 (0.843)	U = 0–3; A = 3–5; HD = 5–7
**CEQ**		
Credibility	18.300 (5.595)	U = 0–13; A = 13–20; HD = 20–27
Expectancy	15.050 (6.004)	U = 0–13; A = 13–20; HD = 20–27
**SUS**	80.3754 (15.558)	U = 0–50; A = 50–72.5; HD = 72.5–100

**Table 3:** Mean and standard deviation (SD) outcomes of Simulator Sickness Questionnarie (SSQ) Pre-experimental test and post-experimental test, and differential values of elderly people. The interpretation of the mean scores for each subscale is provided in the form: N = negligible; M = minimal; S = significant; C = concerning; U = undesirable.

Assessment	Mean (SD) (n = 18)	Score interpretation
**Pre-test SSQ**	3.710 (5.797)	
Nausea	3.710 (5.797)	N<5; M = 5–10; S = 10–15; C = 15–20; U>20
Oculomotor	4.632 (6.946)	
Disorientation	2.320 (5.338)	
**Post-test SSQ**	7.420 (14.062)	
Nausea	7.420 (14.062)	N<5; M = 5–10; S = 10–15; C = 15–20; U>20
Oculomotor	8.422 (13.343)	
Disorientation	6.186 (11.574)	
**Difference**	4.363 (9.350)	
Nausea	3.710 (9.613)	N<5; M = 5–10; S = 10–15; C = 15–20; U>20
Oculomotor	3.79 (8.089)	
Disorientation	3.866 (9.051)	

**Table 4:** Mean and standard deviation (SD) outcomes of Presence Questionnaire (PQ), Satisfaction Questionnaire (GUESS-18) and System usability Scale (SUS) of elderly people. The interpretation of the mean scores for each subscale is provided in the form: U = unacceptable outcome; A = acceptable outcome; HD = highly desirable outcome.

Assessment	Mean (SD) (n = 18)	Normalized mean	Score interpretation
**PQ**			
Realism	29.375 (12.750)	0.699	U = 0–0.5; A = 0.5–0.75; HD = 0.75–1
Possibility to act	12.750 (7.554)	0.531	
Quality of interface	2.500 (2.329)	0.791	
Possibility to examine	11.250 (3.991)	0.625	
Self-evaluation	8.125 (1.807)	0.677	
**GUESS-18**			
Usability	6.625 (0.763)	-	U = 0–3; A = 3–5; HD = 5–7
Narratives	6.406 (0.898)	-	
Play Engrossment	6.218 (1.095)	-	
Enjoyment	5.471 (1.815)	-	
Creative Freedom	5.5004 (1.879)	-	
Audio Aesthetics	5.8214 (1.749)	-	
Personal Gratification	6.937 (0.250)	-	
Social Connectivity	5.500 (1.949)	-	
Visual Aesthetics	6.781 (0.546)	-	
**SUS**	68.472 (18.145)	-	U = 0–50; A = 50–72.5; HD = 72.5–100

### Case study 1: Patients with LLD

#### Intrinsic motivation

The mean value of “interest/enjoyment” subscale (4.593/7.000) is the lowest outcome of all three categories. Despite this result, it is still consider a really good outcome as it indicates that the participants are motivated by PedaleoVR to perform the cycling exercises. While VE and the game is enjoyable, it is understandable to reach monotony at some point during cycling activity, which could affect on the interest aspect. Acknowledging this issue, it may be considered for future iterations of the prototype to include other motor activities that are stimulating for the subject.

The mean value of “value/uselfulness” subscale (4.783/7.000) indicates that the participants perceived PedaleoVR as a useful and valuable tool for their motor functioning recovery. It is also noticed that in some cases participants have given low ratings to the platform, these ratings are attributed to some people’s distrust of these technologies and their lack of habit of using them. But this samples are the less.

#### Credibility/Expectancy

Credibility is associated with logical thinking while expectation is associated with an effective process. Then, the following ideas can be extracted from the results of the Credibility/Expectancy Questionnaire: In terms of credibility, they believe that performing cycling exercise with PedaleoVR can support them in their rehabilitation(18.300±5.595). Besides that, the participants have moderate expectancy (15.050±6.004) that they will improve in their physical functioning by exercising with PedaleoVR.

### Case study 2: Elderly people

#### Simulator sickness questionnaire

SSQ-scores were calculated based on official guidelines [31]. Considering the post-test SSQ results, the scale associated with disorientation had the lowest score (6.186±11.574) followed by the nausea symptomatology scale (7.420±14.062) and finally the oculomotor symptoms scale (8.422±13.343). In general, following aforemention interpretation [32], none of the subscales exceeds 20 points, so it could be said that the VR cycling platform does not cause negative effects. Nevertheless, a recent study [36] suggested administrating the SSQ both before and after the exposure of an experimental condition. Thus, it seemed reasonable to take a baseline to offer more specific insight into the effects of the use of the VR cycling platform by subtracting the values of the previously presented symptoms. After calculating the differences between the pre-test and post-test SSQ, the values of each subscale are still lower (see Table 3). This reinforces the conclusion that the VR cycling platform has no negative effects on elderly people. These results are favourable because it is undesirable to generate adverse effects in the aging population.

#### Presence questionnaire

Regarding the overall QT Score of the patients, a total mean of 71.000/108.000 was obtained with a standard deviation of 23.225. The moderately high score indicates that users are generally satisfied with the VE but it could have been better. This assessment is consistent with the design characteristics of the application, which does not exploit all the auditory and haptic stimulation resources or all the interactive options, which could increase user’ sense of presence. In order to compare the outcomes (see Table 4) of the different subscales with each other, the values have been normalized and represented in a radar plot (Fig 7). Thus, it can be seen that the subscales with the highest mean are the ones corresponding to the quality of the interface with 0.792/1.000 and the realism with 0.701/1.000. While the lowest means subscales are the possibility of acting, with 0.5312/1.000, the possibility to examine with 0.625/1.000, and the self-evaluation performance with 0.677/1.000. Additionally, from the analysis of the raw data, a high correlation was observed between the realism subscale and the ability to act subscale. From this finding, it can be deduced that the visual enhancement of the VR platform with photorealistic graphics could in turn improve the user’s perceived ability to act. Finally, lower values on the scales of possibility to examine and possibility to act are reasonable, as the environment designed for the play objectives of the pedalling activity did not support these possibilities.

#### Game user experience satisfaction scale

Regarding the overall GUESS-18 Score of the patients, Table 3 shows the average of all subscales where the highest values correspond to the subscales of Personal Gratification (6.937/7.000 ± 0.250), Visual Aesthethics(6.781/7.000 ± 0.546), Usability (6.625/7.000 ± 0.763), Narratives (6.406/7.000 ± 0.898) and Play Engrossment (6.218/7.000 ± 1.095). On the other hand, the lowest mean values correspond to the subscales of Enjoyment (5.471/7.000 ± 1.815), Creative Freedom (5.500/7.000 ± 1.815), Social Connectivity (5.500/7.000 ± 1.949) and Audio Aesthetics (5.821/7.000 ± 1.749). The score on the Social Connectivity subscale is particularly noteworthy, as although it is understood that this application has the potential to scale to a multiplayer system and that the social interaction [37, 38] could be an incentive for users. But, drawn from the results, the Social Connectivity aspect does not seem to have been considered relevant by the elderly people themselves. Although the result of the Enjoyment subscale is positive and moderately high, it can also be interpreted in a similar way to the values of the intrinsic motivation subscale carried out with patients with LLD. Since the elderly are also aware and knowledgeable of the benefits of physical activity, it can be considered that the satisfaction reflected also encompasses this self-motivation and the values of the enjoyment subscale hint at this fact. The overall score for all patients is 51.647 over 63, which confirms that participants were very satisfied with the system used. In general, the ratings are consistent with the design of VR cycling platform.

### Usability and feasibility

The results of the SUS from patients with LLD (80.375±15.558) suggest that the ease of use of PedaleoVR is very good. These results were to be expected since the participants had no problems handling the VE. This success is attributed in part to having dedicated an explanation and familiarization phase with the technology prior to the test. Knowing the average age of the participants and predicting their lack of experience with virtual reality technologies, it was deemed necessary to include this previous step in the experimental protocol. This measure did not seem to be sufficient in the case of the elder participants, who did not always show full confidence in the system through the experimental tests, and as a result, the SUS score obtained was almost 12 points lower (68.472±18.145). Even so, both usability ratings were good, if not better in the case of the younger adult group. On the other hand, an interface design was generated that was consistent with the needs of this group of adults. Also, the whole system should be simple and easy to use, it should not generate movement constraints for the patient during pedaling exercise.

## Discussion

The present study aimed first to analyze the usability of the virtual cycling platform by two different population groups with lower limb motor disorders. Secondly, the study sought to answer the prevously stated hypothesis. The results extracted from the standardized questionnaires of each case study (group) are here discussed and summarized.

The IMI questionnaire reflects the subjective experience of the participant with regard to a target activity. This instrument allows the measurement of different aspects of a person’s motivation. Measuring how a system or technology is perceived in terms of usefulness is a way to valid and to predict the technology use, intentions and attitudes towards working with that system, according to Chen et al. [39]. For these reasons, it can be said that this system is positively perceived and valued by adult patients with neuromotor disorders and their acceptance can be presumed. Related to the “Perceived choice” subscale of the IMI (5.281/7.000), the vast majority of them felt likely to perform the cycling activity. This positive predisposition to perform the activity is a key step in the maintenance of physical exercise.

From another point of view, some authors warn that while the IMI questionnaire is able to assess the intensity of motivation, it fails to identify the motivational dimension (intrinsic or extrinsic) [40]. As an example, in the context of PA, participants may indicate that they have enjoyed the activity, not because of the satisfaction of doing the activity per se, but rather because of the extrinsic rewards associated with participation [24, 41]. Translating this concern to our study, it is understood that patients with neurological disorders who attend rehabilitation already have an underlying extrinsic motivation for recovery. Thus, it could be expected that the assessments they make are mainly due to an intrinsic motivation generated by the virtual platform.

However, determining the level to which patients attribute their improvement to the usage of the VR platform can be assess by the CEQ. Regarding the CEQ insights, we can assume that adult neurological patients with LLD are convinced that PedaleoVR is a useful tool that can help them to perform physical activity, but they still need more convincing evidence that PedaleoVR can improve their physical functioning. This could be done by conducting a longitudinal study in which the impact of maintaining over the time the use of PedaleoVR on lower limb motor recovery is observed. Furthermore, providing more information about the positive benefits of performing pedaling activities in improving stability and gait function could improve their expectations. However, it is understood that depending on the severity of the patients’ diagnosis, their expectations of physical improvement are moderate.

Additionally, the satisfaction questionnaire allows to measure the enjoyment of the person during an activity. The results of the Enjoyment or Person Interest subscale of the IMI reported by the LLD patients show values concordant with the results of the Play Engrossment, Enjoyment and Personal Gratification subscales of the GUESS-18 reported by the older adults. On the other hand, the usefulness and value scale of the IMI questionnaire provides information consistent with the credibility subscale of the CEQ. Both subscales reflect the extent to which the participant perceives the use of this tool to be beneficial. Both scales obtained acceptable values.

The sense of presence is an aspect that can significantly influence user motivation. In these terms, the categories of realism and interface quality are the most highly rated. Similarly, the visual aesthetics category of the GUESS-18 questionnaire is highly rated. In fact, all subscales of GUESS-18 scored on average between 5.4 and 7 points. These highly desirable values reveal the high user satisfaction with the platform. Although the results of the motivation, credibility and satisfaction scale show that users find the use of this platform rewarding, motivating, interesting and potentially valuable, it is necessary to re-evaluate the effect of this motivation in the long term. For there may be a novelty factor that alters this perception of motivation and therefore the actual engagement may decline over repeated use. This has also been noted in other similar studies [24].

In general, all Nausea, Oculomotor and Disorientation subscales of SSQ scored below 10 points, considering the adverse effects to be minimal. Even though, the value of each subscale is also shown in Table 3 as the difference in scores between the pre-exposure and post-exposure measures and, in this case, the values are negligible. Compared to other studies [23, 24], the adverse effects measured are lower than those measured by previous studies. This may be attributed to the fact that the aesthetics of the virtual environment and the design of the platform are more sensitive to avoiding general user discomfort. Regarding usability, both populations have been explicitly consulted about this aspect of the platform. It can be concluded that for the adult population (whose mean age is 85.16 with standard deviation = 5.93) the ease of use is rated as adequate, while the population of patients with LLD (whose mean age is 61.10 with standard deviation = 12.62) rates the ease of use of the platform as excellent. We can conclude that both populations rated the usability of the platform positively, with a higher rating from the adult population with LLD.

### PedaleoVR design aspects

It was essential to achieve a prototype that was consistent with these two characteristics. First, in order to make it easy to handle for these patients, whose neurological damage could also affect the upper limb, the use of VR controllers was dismissed. In line with improvements in hand recognition software, which is increasingly being used in the field of rehabilitation applications [42], we implemented and hand-tracking-based interactions with the VE. Second, the use of inertial sensors for controlling exergames has become used the most in virtual training tools [43, 44] as they are getting cheaper, their accuracy is increasing and gesture recognition is improving [45]. In addition to these reasons, the ENLAZA™ sensor is included, with the primary intention of incorporating a non-obstructive pedal movement capture system during pedaling that is adaptable for all patients and all stationary pedaling stations. As a result, it can be stated for the usability assessment of PedaleoVR, that it is perfectly feasible and easy to use tool for elderly people and patients with LLD.

### Methodological aspects

As the present research is a usability and feasibility study, only descriptive statistics can be performed. For these reason, our results have to be handle with the upmost care. Findings of this study provide a context for the use of PedaleoVR in two different populations, and describe how the usage this novel system is perceived and accepted. However, analyzing the success of PedaleoVR as a tool to enhance pedaling exercise and its effects in adult patients with LLD requires further studies, as well as its potential effects in lower limb strengthen in elderly people. Nevertheless, the motivation and satisfaction outcomes agree with the reviewed studies which showed that exergames intervention groups were more motivated to exercise [46] and found the training more appealing than traditional exercises [47].

### Limitations

This study has several limitations. First, given the descriptive nature of the study, we did not consider the current routine therapies performed by the participants. Therefore, it is possible that participants had different physical activity baselines, different habits of using technological tools, and different expectations regarding the platform presented.

Secondly, in the group of neurological patients, we can differentiate between a group of younger participants around 50 years of age and another group of older participants around 70 years of age. In the case of the group of older people, we find a group around 85 years of age. If we could have had a larger sample of participants in both cases, all the metrics could be analysed according to different age groups. However, due to this limitation, it has not been possible to carry out this characterisation, with the exception of SUS outcomes, where differences have been observed between the two groups, which presumably could be due to the difference in age and familiarity with the technology.

As a final observation, it is also worth considering that, pedaling exercise can be exhausting for those participants who are not in suitable physical condition to undertake the effort. This issue can compromise the enjoyment of the pedaling activity. Therefore, having a pedaling assistance system could be useful for these patients to avoid generating an initial demotivating due to lack of adaptation of the system.

## Conclusion

Our research describes the core aspects of a virtual reality platform based on a standalone system for the promotion of pedalling activity in an immersive environment. The findings allow us to address enhancements and future designs of VE for older adults and patients with LLD. In overall conclusion, all participants agreed on great aesthetics of the VE and the VR platform design in terms of usability. These aspects could promote the enjoyment of the activity and personal gratification, which would also be contributing to the participant’s motivation. Moreover, it has also been verified that the platform does not generate adverse effects due to the cybersickness of virtual reality in static activities. This evaluation has been carried out in an adult population and no cases of rejection of the technology for these reasons have been reported. Finally, further studies should explore the extent to which intrinsic motivation is maintained in the long-term. As a future direction it is considered that the addition of sound feedback, as well as performance scoring, may improve the user experience and, consistently, the engagement in exercise.

## Supporting information

S1 File Datasheet URL. (RAR)

S2 File Research work report. (PDF)

S1 Checklist TREND statement checklist. (PDF)

## References

[1] Group GNDC. Global, regional and national burden of neurological disorders during:1990-2015: a systematic analysis for the Global Burden of Disease Study 2015. The Lancet Neurology. 2017;16(11):877–897. doi: 10.1016/S1474-4422(17)30299-5 28931491 PMC5641502

[2] Pollock A, Baer G, Campbell P, Choo P, Forster A, Morris J, et al. Physical rehabilitation approaches for the recovery of function and mobility following stroke. Cochrane Database. 2014;(4). doi: 10.1002/14651858.CD001920.pub3 24756870 PMC6465059

[3] Belda-Lois JM, Mena-del Horno S, Bermejo-Bosch I, Moreno JC, Pons JL. Rehabilitation of gait after stroke: a review towards a top-down approach. Journal of NeuroEngineering and Rehabilitation. 2011;8(1):1–20. doi: 10.1186/1743-0003-8-66 22165907 PMC3261106

[4] Moreno JC, Barroso F, Farina D, Gizzi L, Santos C, Molinari M, et al. Effects of robotic guidance on the coordination of locomotion. Journal of NeuroEngineering and Rehabilitation. 2013;10(79). doi: 10.1186/1743-0003-10-79 23870328 PMC3724716

[5] Marchal-Crespo L, Tsangaridis P, Obwegeser D, Maggioni S, Riener R. Haptic Error Modulation Outperforms Visual Error Amplification When Learning a Modified Gait Pattern. Frontiers in Neuroscience. 2019;13(60). doi: 10.3389/fnins.2019.00061 30837824 PMC6390202

[6] Statistics E. Explained, Population structure and ageing; 2015.

[7] Gregersen CS, Hull M. Non-driving intersegmental knee moments in cycling computed using a model that includes three-dimensional kinematics of the shank/foot and the effect of simplifying assumptions. Journal of biomechanics. 2003;36(6):803–813. doi: 10.1016/S0021-9290(03)00014-9 12742448

[8] of Health NI, et al. NIH consensus development panel on osteoporosis prevention, diagnosis, and therapy, March 7-29, 2000: highlights of the conference. South Med J. 2001;94(6):569–73. doi: 10.1097/00007611-200194060-00004 11440324

[9] Valenzuela PL, Castillo-García A, Morales JS, Izquierdo M, Serra-Rexach JA, Santos-Lozano A, et al. Physical exercise in the oldest old. Comprehensive physiology. 2011;9(4):1281–1304. 10.1002/cphy.c190002 31688965

[10] Larsson L, Degens H, Li M, Salviati L, Lee YI, Thompson W, et al. Sarcopenia: aging-related loss of muscle mass and function. Physiological reviews. 2019;99(1):427–511. doi: 10.1152/physrev.00061.2017 30427277 PMC6442923

[11] Mazzocchio R, Meunier S, Ferrante S, Molteni F. Cycling, a tool for locomotor recovery after. NeuroRehabilitation. 2008;23:67–80. doi: 10.3233/NRE-2008-23107 18356590

[12] Lazoura O, Papadaki PJ, Antoniadou E, Groumas N, Papadimitriou A, Thriskos P, et al. Skeletal and body composition changes in hemiplegic patients. Journal of Clinical Densitometry. 2010;13(2):175–180. doi: 10.1016/j.jocd.2010.01.008 20347365

[13] Patterson SL, Forrester LW, Rodgers MM, Ryan AS, Ivey FM, Sorkin JD, et al. Determinants of walking function after stroke: differences by deficit severity. Archives of physical medicine and rehabilitation. 2007;88(1):115–119. doi: 10.1016/j.apmr.2006.10.025 17207686

[14] Sugiyama T, Liew SL. The Effects of Sensory Manipulations on Motor Behavior: From Basic Science to Clinical Rehabilitation. J Mot Behab. 2017;49(1):67–77. doi: 10.1080/00222895.2016.1241740 27935445 PMC6124483

[15] Perez MA, Lungholt BK, Nyborg K, Nielsen JB. Motor skill training induces changes in the excitability of the leg cortical area in healthy humans. Experimental Brain Research. 2004;159(2):197–205. doi: 10.1007/s00221-004-1947-5 15549279

[16] Waliño-Paniagua CN, Gómez-Calero C, Jiménez-Trujillo MI, Aguirre-Tejedor L, Bermejo-Franco A, Ortiz-Gutiérrez RM, et al. Effects of a game-based virtual reality video capture training program plus occupational therapy on manual dexterity in patients with multiple sclerosis: a randomized controlled trial. J Healthc Eng. 2019;. doi: 10.1155/2019/9780587 31178989 PMC6501239

[17] Webster D, Celik O. Systematic review of Kinect applications in elderly care and stroke rehabilitation. J Neuroeng Rehabil. 2014;11(108). doi: 10.1186/1743-0003-11-108 24996956 PMC4094409

[18] Molina KI, Ricci NA, de Moraes SA, Perracini MR. Virtual reality using games for improving physical functioning in older adults: a systematic review. J Neuroeng Rehabil. 2014;11(156). doi: 10.1186/1743-0003-11-156 25399408 PMC4247561

[19] Itakussu EY, Valenciano PJ, Trelha CS, Marchiori L. Benefits of Exercise Training with NINTENDO^®^ Wii for Healthy Elderly Population: Literature Review. CEFAC. 2015;17(3).

[20] De Boissieu P, Denormandie P, Armaingaud D, Sanchez S, Jeandel C. Exergames and elderly: a non-systematic review of the literature. Eur Geriatr Med. 2017;8(2):111–117. doi: 10.1016/j.eurger.2017.02.003

[21] C G et al. Effects of virtual reality associated with serious games for upper limb rehabilitation in patients with multiple sclerosis: randomized controlled trial. Journal of NeuroEngineering and Rehabilitation. 2020;17(10):2–10. 32660604 10.1186/s12984-020-00718-x PMC7359450

[22] Knippenberg E, Timmermans A, Palmaers S, Spooren A. Use of a technology-based system to motivate older adults in performing physical activity: a feasibility study. BMC Geriatrics. 2021;21(81). doi: 10.1186/s12877-021-02021-3 33509098 PMC7841896

[23] Katsigiannis S, Willis R, Ramzan N. A qoe and simulator sickness evaluation of a smart-exercise-bike virtual reality system via user feedback and physiological signals. IEEE Transactions on Consumer Electronics. 2018;65(1):119–127. doi: 10.1109/TCE.2018.2879065

[24] Høeg ER, Bruun-Pedersen JR, Cheary S, Andersen LK, Paisa R, Serafin S, et al. Buddy biking: a user study on social collaboration in a virtual reality exergame for rehabilitation. Virtual Reality. 2021; p. 1–18.

[25] Rojo A, Raya R, Moreno JC. Virtual reality application for real-time pedalling cadence estimation based on hip ROM tracking with inertial sensors: a pilot study. Virtual Reality. 2022; p. 1–15.

[26] Raya R, Garcia-Carmona R, Sanchez C, Urendes E, Ramirez O, Martin A, et al. An Inexpensive and Easy to Use Cervical Range of Motion Measurement Solution Using Inertial Sensors. Sensors. 2018;18. doi: 10.3390/s18082582 30087258 PMC6111246

[27] Costa V, Ramírez O, Otero A, Muñnoz-García D, Uribarri S, Raya R. Validity and reliability of inertial sensors for elbow and wrist range of motion assessment. PeerJ. 2020;8. doi: 10.7717/peerj.9687 32864213 PMC7427560

[28] MOTOmed R. MOTOmed viva 2. Short instructions;. Available from: https://www.medimotion.co.uk//files/manual_viva2.pdf.

[29] Richard MR, Edward LD. Intrinsic motivation and self-determination in human behavior. American Psychologist. 2000;55(1):688–78.

[30] Devilly Grant, Borkovec Thomas. Psychometric properties of the Credibility/Expectancy Questionnaire. Journal of Behavior Therapy and Experimental Psychiatry. 2000;31:73–86. doi: 10.1016/S0005-7916(00)00012-4 11132119

[31] Kennedy RS, Lane NE, Berbaum KS, Lilienthal MG. Simulator sickness questionnaire: An enhanced method for quantifying simulator sickness. The international journal of aviation psychology. 1993;3(3):203–220. doi: 10.1207/s15327108ijap0303_3

[32] Stanney KM, Kennedy RS, Drexler JM. Cybersickness is not simulator sickness. In: Proceedings of the Human Factors and Ergonomics Society annual meeting. vol. 41. SAGE Publications Sage CA: Los Angeles, CA; 1997. p. 1138–1142.

[33] Witmer BG, Singer MJ. Measuring presence in virtual environments: A presence questionnaire. Presence. 1998;7(3):225–240. doi: 10.1162/105474698565686

[34] Keebler JR, Shelstad WJ, Smith DC, Chaparro BS, Phan MH. Validation of the GUESS-18: a short version of the Game User Experience Satisfaction Scale (GUESS). Journal of Usability Studies. 2020;16(1):49.

[35] Brooke J. SUS—A quick and dirty usability scale. Usability Eval ind. 1996;189(194):4–7.

[36] Bimberg P, Weissker T, Kulik A. On the usage of the simulator sickness questionnaire for virtual reality research. In: 2020 IEEE Conference on Virtual Reality and 3D User Interfaces Abstracts and Workshops (VRW). IEEE; 2020. p. 464–467.

[37] Saracchini R, Catalina C, Bordoni L. Tecnología asistencial móvil, con realidad aumentada, para las personas mayores = A Mobile Augmented Reality Assistive Technology for the Elderly. Tecnología asistencial móvil, con realidad aumentada, para las personas mayores = A Mobile Augmented Reality Assistive Technology for the Elderly. 2015; p. 65–83.

[38] Triandafilou KM, Tsoupikova D, Barry AJ, Thielbar KN, Stoykov N, Kamper DG. Development of a 3D, networked multi-user virtual reality environment for home therapy after stroke. Journal of neuroengineering and rehabilitation. 2018;15(1):1–13. doi: 10.1186/s12984-018-0429-0 30290777 PMC6173932

[39] Chen T, Bhattacharjee T, Beer JM, Tin LH, Hackney ME, Rogers WAea. Older adults’ acceptance of a robot for partner dance-based exercise. PLoS One. 2017;12(10). doi: 10.1371/journal.pone.0182736 29045408 PMC5646767

[40] Markland D, Hardy L. On the factorial and construct validity of the Intrinsic Motivation Inventory: Conceptual and Operational concerns. Research Quarterly for Exercise and Sport. 1997;68(1):20–32. doi: 10.1080/02701367.1997.10608863 9094760

[41] Ryan RM, Mims V, Koestner R. Relation of reward contingency and interpersonal context of intrinsic motivation: A review and test using cognitive evaluation theory. J Personality and Social Psychology. 1983;45:736–750. doi: 10.1037/0022-3514.45.4.736

[42] Lange B, Koenig S, Chang CY, McConnell E, Suma E, Bolas M, et al. Designing informed game-based rehabilitation tasks leveraging advances in virtual reality. Disabil Rehabil. 2012;34:1863–1870. doi: 10.3109/09638288.2012.670029 22494437

[43] Saposnik G, Teasell R, Mamdani M, Hall J, McIlroy W, Cheung D, et al. Effectiveness of Virtual Reality Using Wii Gaming Technology in Stroke Rehabilitation. A Pilot Randomized Clinical Trial and Proof of Principle. Stroke. 2010;41:1477–1484. doi: 10.1161/STROKEAHA.110.584979 20508185 PMC4879973

[44] Kosse N, Caljouw S, Vuijk P, CJC L. Exergaming: interactive balance training in healthy community-dwelling elderly. Journal of Cyber Therapy & Rehabilitation. 2011;4:399–407.

[45] Patel S, Park H, Bonato P, Chan L, Rodgers M. A review of wearable sensors and systems with application in rehabilitation. J NeuroEngineering Rehabilitation. 2012;9(21). doi: 10.1186/1743-0003-9-21 22520559 PMC3354997

[46] Fitzgerald D, Trakarnratanakul N, Smyth B, Caulfield B. Effects of a wobble board-based therapeutic exergaming system for balance training on dynamic postural stability and intrinsic motivation levels. J Orthop Sports Phys Ther. 2010;40:11–19. doi: 10.2519/jospt.2010.3121 20044704

[47] Betker A, Szturm T, Moussavi Z. Development of an interactive motivating tool for rehabilitation movements. In: 3, editor. Conf Proc IEEE Eng Med Biol Soc; 2005. p. 2341–2344. 10.1109/IEMBS.2005.1616935 17282704

