# Supplementary material for: PedaleoVR: Usability study of a virtual reality application for cycling exercise in patients with lower limb disorders and elderly people
Source: PLoS One. 2023 Feb 22;18(2):e0280743. doi: 10.1371/journal.pone.0280743 (PMC9946226; doi:10.1371/journal.pone.0280743)
Supplement: S2 File — (PDF) [file pone.0280743.s002.pdf]

Project/Research work report

1. Title: Development and study of an interactive platform and an electronic pedalling system for functional rehabilitation of the lower limb.

2. Objectives

The objective of this research is to combine the use of inertial systems with virtual reality in pedalling exercises in a pilot study with subjects with ataxia or hemiparesis. In particular, the aim is to evaluate the validity of the system as a physical training tool for pedalling exercises aimed at providing motivational visual stimuli and biofeedback based on pedalling cadence to improve the exercise experience and promote adherence to the subject's treatment.

The proposed research is of a longitudinal experimental cohort type, as two groups of participants are proposed: the experimental group will perform pedalling exercises while using the virtual reality system and the control group will perform pedalling exercises without using the virtual reality system. Thus, the factor that varies between the two groups is the use of virtual reality.

Primary objectives:

- To study the estimation of pedalling cadence performed by the system/platform in subjects with hemiparesis or ataxia.
- To study the impact of the platform on the motor improvement of the lower limb of the participating subjects. To compare the results between the experimental group and the control group.
- To study the characteristics of usability, credibility and intrinsic motivation of the platform.

3. Analysis of potential benefits and risks of the study.

The use of inertial systems and reality devices have a low incidence of adverse reactions, falls or dizziness. In order to minimise these risks, the experiment will be supervised at all times by a specialist in the use of such technology, who will be in charge of accompanying and assisting the participant during the test. Compared to the traditional rehabilitation programme, there are no additional risks associated with the use of virtual reality devices during the task.

The main benefit of participating in the study would be a greater range of motion in the hips and knees and possible improvement in gait functions as a result of the pedalling exercise.

4. Material and Methods

The clinical trials with patients will take place in the facilities of the Lescer Centre, with the informed consent of the patients and the supervision and collaboration of the physiotherapists of the neurorehabilitation centre.

The volunteer subjects to be recruited will be patients with ataxia or hemiparesis in the lower limb of the Lescer Centre, aged between 18 and 85 years, of heights between 150 and 195 centimetres, of both sexes and whose condition and clinical history rule out any incompatibility with the use of a virtual reality system. These data will be pseudo-anonymised (coded).

Data relating to the pedalling kinematics of the volunteer subjects will be managed in real time. This data will be captured by an inertial sensor system. This data shall not be extracted after the end of the session, nor shall it be stored or studied. The pedalling kinematics data is only used in real time as input to the visual biofeedback system. This biofeedback system has already been validated in a previous study with healthy subjects.

Data on the usability of the tool, reliability and credibility of the tool and motivation generated by the tool will be obtained by means of approved questionnaires. These data are ideal to meet the objectives of the study by providing information for the validation of the virtual reality tool, which is crucial for the design of the strategy of visual feedback to the volunteer subject and the characterisation of the effective cadence of their pedalling.

The non-invasive and passive nature of the data to be collected during the experiment makes it perfect for use on humans and presents no risk to the safety of the volunteer subject.

4.1. Sample size

The data analysis models will be 2-group repeated measures models and analysis of variance pending the probability distributions and longitudinal effect.

The sample size has been calculated using the software tool G*Power 3.1.9.7. The extract of the calculation is included below:

F tests - ANOVA: Repeated measures, between factors

Analysis: A priori: Compute required sample size

Input: Effect size f = 0.7

α err prob = 0.05

Power (1-β err prob) = 0.95

Number of groups = 2

Number of measurements = 4

Corr among rep measures = 0.5

Output: Noncentrality parameter λ = 15.6800000

Critical F = 4.4138734

Numerator df = 1.0000000

Denominator df = 18.0000000

Total sample size = 20

Actual power = 0.9625393

Ideally, assuming an effect size of 0.7, a sample of 20 subjects is required for the study. In this case, the analysis of these data would have a statistical power of 0.96.

However, the sample size will depend on the number of volunteers that the Lescer Centre team has available, trying to reach a minimum sample of N=20 (n=10 for each group) sufficient to carry out the relevant descriptive statistics.

4.2. Protocol

The procedure will be carried out in the following phases, which are detailed below:

Phase 1: **Enrolment and participant information.**

Participants are recruited according to the following inclusion and exclusion criteria:

Inclusion Criteria

- Patients with Ataxia or Hemiparesis.
- Partial lower limb motor function.
- Sign and understand the informed consent form.

Exclusion Criteria

- Presence of any electronic device implanted in the head.
- Presence of stiffness in the joints of the lower extremity.
- Suffering from vertigo with dizziness.
- History of previous lower limb surgery within the last 6 months.

The subject is informed about the experiment in which he/she is going to participate, its duration and the technical characteristics to be used in the study. **The data to be collected is explained to the subject in the form of standardised questionnaires and metrics for functional assessment of gait and joint range, and at no time personal data. If the subject is suitable for participation, he/she will be asked to sign the corresponding informed consent form.** After confirmation of the participant, he/she will be randomly assigned to the control or experimental group.

Finally, 3 appointments with the patient will be arranged in accordance with the general experimental schedule. Between appointments, at least on consecutive days and at most within 36 hours, in order to avoid losing the effect of the continuous training. This maximum time frame is considered as it is understood that most participants will not attend sessions at the clinic during weekends. Ideally, patients will be seen every 48 hours after the last session, i.e. on alternate days (e.g. Monday-Wednesday-Friday).

Phase 2: **Instrumentation familiarisation practice.** These will be aimed at acquiring basic skills in the use of the virtual reality environment synchronised with the pedalling task.

Phase 3: **Experimentation and data acquisition.**

A.1. Pre-assessment: The participant's motor skills are assessed based on the application of physical assessment metrics (Functional Gait Scale "Timed Up & Go", TUG) [1] carried out by the physician. Participants with no visual defects, mild cognitive impairment and lower limb motor skills are selected.

A.2. Virtual Reality Platform Usability Validation Session for Patients with Ataxia or Hemiparesis (Approx. 40 minutes)

Non-invasive intervention: A pedalling exercise is performed on a bicycle or static pedalling station synchronising the physical activity with the visual feedback of the virtual reality application. Two pedalling sets of 5 minutes each are performed, with 1 minute rest between sets.

A.3. Objective Evaluation: After the exercise task of the first session, the participant is asked to rate the usability: System Usability Rating Scale (SUS) [2], Credibility and Expectancy Questionnaire (CEQ) [3] and Intrinsic Motivation Inventory (IMI) [4] of the virtual reality application.

A.4. Subsequent evaluation: The participant's motor skills are evaluated after the three pedalling sessions, based on the application of physical assessment metrics carried out by the physician.

Summary of the procedures to be carried out during the study (Phase 3):

The protocol foresees three experimental sessions spaced in time. The session begins with a phase of explanation and clarification of the protocol to be followed (approximately 5 minutes) and the use of the pedalling system, for both groups.

In the case of the experimental group, there will also be a brief explanation of the use of the virtual reality device and the inertial sensor. The participant will then be fitted with the virtual reality device, as well as the motion capture system consisting of a single inertial sensor placed on the thigh using an adjustable elastic band. In the case of patients with hemiparesis, the inertial sensor will be placed on the most affected side. This criterion shall be applied in all sessions (3). By default, ataxia patients will have the inertial sensor placed on the right thigh.

Once the participant is ready, a series of test pedal strokes will be performed to familiarise them with the methodology to be followed during the test. In the case of the experimental group, participants will perform this test to familiarise themselves with the visual feedback system. In both cases the patient will be instructed to pedal at a comfortable speed.

Once the subject has become familiar with the procedure, and after a rest interval of approximately 5 minutes, the experimental phase will begin, in which the same conditions will be studied for all participants in all sessions:

Two sets/exercises of 5 minutes pedalling at constant speed are performed, with recovery time of 1 minute between sets.

At the end of the physical task, the usability, credibility and intrinsic motivation assessment questionnaires will be completed.

Phase 4: **Data analysis, interpretation and dissemination.**

A. Data will be processed and statistically analysed using mathematical models equivalent to those used in similar studies in the literature.

B. The interpretation of the results will be used for the development of an alternative and complementary physical activity promotion tool based on pedalling exercises for patients with ataxia and hemiparesis. Therefore, it will be disseminated in scientific journals, as well as in seminars or scientific events, always keeping the anonymity of the volunteers participating in the study.

C. In accordance with clause Eight. Publications, of the Collaboration Agreement between the Agencia Estatal Consejo Superior de Investigaciones Científicas, (Spanish National Research Council, CSIC) M.P., the Universidad San Pablo CEU and the company Werium Assistive Solutions for the financing of the industrial doctorate project in which this project is framed, the use of the partial or final results for publication or dissemination will be managed between the parties: Werium Assistive Solutions and CSIC.

D. The records obtained in the experimental phase will be organised in a coded database, together with the demographic data. In accordance with clause eight of the collaboration agreement between the three parties: CSIC, Werium Assistive Solutions and San Pablo CEU University, the data from the evaluation of the system by means of questionnaires will be kept under the custody of the researcher in a CSIC data repository, safeguarding its confidentiality.

4.3. Materials

4.3.1. Virtual Reality system

This VR system is based on the communication of pedalling data and information around the control computer. The data transmission from the inertial sensors to the Oculus Quest viewer is established via Bluetooth. In this way, this interface supports the data processing of pedalling cycles, speed and distance travelled by each user and the transmission of these values to the immersive scenarios. The Virtual Reality scenario generated for this therapy consists of controlling the forward movement of an aeroplane by pedalling. Thus, the patient is placed inside the plane's cockpit and visualises the session data on the plane's control panel.

4.3.2. Inertial Sensors

The motion capture system for pedal kinematic analysis to be used is the ENLAZA sensor from Werium Assistive Solutions, due to the proven reliability of its ROM measurements at the cervical (7), wrist and elbow joints (6). The ENLAZA sensor module contains an Inertial Measurement Unit (IMU) with 9 degrees of freedom, which integrates a 3-axis accelerometer, a 3-axis gyroscope and a 3-axis magnetometer. The sensor also includes a Bluetooth module (2.4 GHz) through which the IMU data is sent to the VR device.

4.3.3. Visual Biofeedback System

The intelligent system implemented in both systems evaluates the average pedalling speed of the last 3 seconds with respect to the set speed, every second. A threshold of acceptance of the instantaneous speed is set at ±15% of the set speed. Higher values are considered too fast and lower values too slow, so pop-up messages are issued to moderate or increase the pedalling rate accordingly. Motivational messages are displayed when the user maintains an adequate pace.

5. Research Planification

Activity		S1	S2	S3	S4	S5	S6
Participants involvement	Lescer Clinician	■					
Sessions' planning	Lescer Clinician	■					
Initial Physical Assessment	Lescer Clinician and PhD Student CEU		■				
Intervention Program	Lescer Clinician and PhD Student CEU		■	■	■		
Physical Assessment	Lescer Clinician and PhD Student CEU				■		
Data collection	PhD Student CEU				■		
Analysis of results	PhD Student CEU					■	■

In order to carry out the study, it will be essential to have the collaboration of the physiotherapist of the Lescer Centre responsible for the management of patients participating in the study. This person will manage the first phases of recruitment of participants who meet the inclusion and exclusion criteria and the planning of the sessions according to the agenda of the Lescer Centre. The tasks of physical assessments and implementation of the pedalling intervention programme will be carried out jointly by the Trainee Researcher (FI) and the specialist responsible for the Lescer Centre.

Finally, the tasks of data collection, coding and data processing will be carried out by the Researcher in Training.

6. Bibliography

[1] Podsiadlo D, Richardson S. (1991) The Timed "Up & Go": A Test of Basic Functional Mobility for Frail Elderly Persons. Journal of the American Geriatrics Society. 39(2): 142-8

[2] J. Brooke. (1996) SUS - A quick and dirty usability scale. Usability Eval. Ind., 189 (194), pp. 4-7.

[3] Devilly, Grant & Borkovec, Thomas. (2000). Psychometric properties of the Credibility/Expectancy Questionnaire. Journal of Behavior Therapy and Experimental Psychiatry. 31. 73-86. 10.1016/S0005-7916(00)00012-4

[4] Ryan RM. 1982. Control and information in the intrapersonal sphere: an extensión of cognitive evaluation theory. J Pers Soc Psychol 43:450-61.

[5] Knippenberg, Els & Timmermans, Annick & Palmaers, Steven & Spooren, Annemie. (2020). Use of a technology-based system to motivate older adults in performing physical activity: a feasibility study.. 10.21203/rs.3.rs-53576/v2.

[6] Costa V, Ramírez Ó, Otero A, Muñoz-García D, Uribarri S, Raya R. 2020. Validity and reliability of inertial sensors for elbow and wrist range of motion assessment. *PeerJ* 8:e9687

[7] Raya, R.; Garcia-Carmona, R.; Sanchez, C.; Urendes, E.; Ramirez, O.; Martin, A.; Otero, A. (2018) An Inexpensive and Easy to Use Cervical Range of Motion Measurement Solution Using Inertial Sensors. *Sensors 18*, 2582. https://doi.org/10.3390/s18082582
